# Independent associations of fatigue with cognitive function and quality of life in multiple sclerosis: a cross-sectional study

**DOI:** 10.3389/fpsyg.2026.1833477

**Published:** 2026-05-05

**Authors:** Caterina Formica, Lilla Bonanno, Laura Culicetto, Antonino Lombardo Facciale, Edoardo Sessa, Carmela Rifici, Angelo Quartarone, Silvia Marino, Rocco Salvatore Calabrò, Desirèe Latella

**Affiliations:** IRCCS Centro Neurolesi Bonino Pulejo, Messina, Italy

**Keywords:** cognition, fatigue, multiple sclerosis, neurological disability, quality of life

## Abstract

**Introduction:**

The aim of the study is to investigate the association between fatigue, cognitive impairment, and overall QoL in individuals with Multiple Sclerosis (MS).

**Methods:**

Fifty-one patients with MS with a confirmed diagnosis of relapsing–remitting multiple sclerosis (RRMS), according to established diagnostic criteria were enrolled. Data were collected at a single time point to examine associations between fatigue, cognitive functioning, neurological disability, and health-related quality of life (QoL). All participants were submitted to a standardized neurological examination, Fatigue symptoms were assessed using the Fatigue Severity Scale (FSS), Modified Fatigue Impact Scale (MFIS) and Fatigue Scale for Motor and Cognitive Functions (FSMC). Cognitive profiles were evaluated with the Brief Repeatable Battery of Neuropsychological Tests (BRB-N), overall Quality of Life with the Multiple Sclerosis Quality Ff Life (MSQOL-54) and the Expanded Disability Status Scale (EDSS) to evaluate the disability status.

**Results:**

Our findings indicate a significative correlation between general FATIGUE and multiple QoL outcomes. Specifically, fatigue was strongly correlated with Health Perception (*ρ* = 0.63, *p* < 0.001), Overall Quality of Life (*ρ* = 0.65, *p* < 0.001), Role Physical Limitation (*ρ* = 0.43, *p* = 0.002), and Role Emotional Limitation (*ρ* = 0.37, *p* = 0.007), and is also related to specific aspects of cognitive functioning as well as Symbol Digit Modalities Test (SDMT) (*r* = −0.38, *p* = 0.006) and Selective Reminding Test – Delayed (*r* = −0.36, *p* = 0.01).

**Discussion:**

These results contribute to a more comprehensive understanding of disease burden in MS, highlighting fatigue as a key factor that interacts with multiple dimensions of patient functioning.

**Clinical trial registration:**

Identifier (NCT 05783999).

## 1 Introduction

Multiple sclerosis (MS) is a chronic neurodegenerative disease characterized by inflammatory demyelination and axonal damage within the central nervous system (CNS). Depending on the cerebral areas involved, a wide range of neurological symptoms may occur, affecting visual, motor, sensory, and autonomic systems. In addition to physical impairments, MS is frequently associated with psychopathological symptoms such as fatigue, depression, cognitive dysfunction, and sleep disturbances, all of which substantially impact patients’ quality of life (QoL) (Amato et al., 2001; Lobentanz et al., 2004). Among these symptoms, fatigue is consistently reported as one of the most disabling and burdensome, affecting up to 92% of individuals with MS (Bol et al., 2009; Janardhan and Bakshi, 2002; Krupp, 2003). Fatigue in MS is not limited to physical tiredness but often manifests as mental fatigue, characterized by difficulties in sustaining attention and maintaining cognitive effort over time (Bryant et al., 2004; Schwid et al., 1999). This persistent lack of mental energy has been linked to reduced participation in daily activities, impaired social functioning, and diminished psychological well-being (Krause et al., 2013).

Over recent years, increasing attention has been devoted to the relationship between fatigue and cognitive functioning in MS, with the aim of clarifying the role of fatigue in cognitive impairment and physical disability (Cehelyk et al., 2019; Taranu et al., 2025). However, findings remain inconsistent. Several studies have reported no independent association between fatigue and neurological disability as measured by the EDSS (Bakshi et al., 2000; Biberacher et al., 2018; Ghajarzadeh et al., 2013). Instead, perceived fatigue appears to be more closely related to depressive symptoms than to objective neurological impairment (Bakshi et al., 2000). Both cross-sectional and longitudinal studies have identified depression as a frequent covariate of fatigue, suggesting that fatigue may be relatively independent of physical disability but strongly associated with affective factors. However, these findings point to the multidimensional nature of fatigue, indicating that, particularly in the case of perceived fatigue, multiple factors contribute to its underlying mechanisms and are not always associated with levels of disability, which reflect a more objective measure of the symptom. This overlap points to shared underlying mechanisms, potentially involving psychological processes or damage to specific neuroanatomical pathways implicated in mood and motivation (Andreasen et al., 2010; Fernández-Muñoz et al., 2018; Patrick et al., 2009).

In parallel, a growing body of evidence has focused on cognitive performance fatigability during sustained mental tasks (Berard et al., 2018; Claros-Salinas et al., 2013; Krupp and Elkins, 2000). The factors contributing to cognitive decline in MS remain poorly understood, partly due to heterogeneity in study designs and the frequent simultaneous assessment of physical and cognitive performance (Cehelyk et al., 2019; Wolkorte et al., 2015). Although fatigue has been extensively studied in MS populations, considerable variability persists in the literature regarding its relationship with cognitive functioning, disability level, and overall QoL (Eizaguirre et al., 2020).

Clarifying the role of fatigue associated to cognitive dysfunction and QoL is therefore crucial, particularly given its implications for treatment responsiveness, clinical monitoring, and the long-term management of MS (Bol et al., 2009). Although fatigue in MS has been extensively investigated, previous findings remain heterogeneous, and studies have often examined its relationship with cognition, disability, or QoL separately. Less attention has been devoted to evaluating these dimensions together within the same clinically homogeneous Relapsing–Remitting Multiple Sclerosis (RRMS) cohort and to clarifying whether fatigue is associated with selected cognitive and QoL outcomes beyond neurological disability. Therefore, the present study aimed to investigate the independent associations of fatigue with cognitive performance and overall QoL in individuals with RRMS, using multiple fatigue measures. In addition, an exploratory stratified analysis by disability level was performed to examine whether these relationships differed according to neurological disability severity, in order to better define the multidimensional clinical burden of fatigue.

## 2 Materials and methods

### 2.1 Study design and participants

This observational, cross-sectional study was conducted at the Multiple Sclerosis outpatient clinic of the IRCCS Centro Neurolesi “Bonino Pulejo” in Messina, Italy. The study sample consisted of fifty-one participants, with a mean age of 46.37 (SD = 12.39). Based on the EDSS score, participants were divided into two groups: EDSS ≤5 (*n* = 27) and EDSS >5 (*n* = 24). The EDSS ≤5 group had a mean age of 39.14 (SD = 10.70), whereas the EDSS >5 group had a mean age of 51.86 (SD = 10.76). The sample included 25 male and 26 famale (EDSS ≤5 group = 16 M, 17F; EDSS >5 group = 9 M, 9F). We recruited patients with confirmed diagnosis of RRMS according to established diagnostic criteria, participants had a mean disease duration of 8.7 (SD = 5.2), for group with EDSS ≤5 mean 6.6 (SD = 3.9), group with EDSS >5 mean 12.0 (SD = 5.4)The study protocol was approved by the local Ethics Committee of the IRCCS Centro Neurolesi “Bonino Pulejo” (approval number U74/21), and all participants provided written informed consent in accordance with the Declaration of Helsinki.

Data were collected at a single time point to examine associations between fatigue, cognitive functioning, neurological disability, and health-related QoL. However, it is important to note that the QoL instrument adopted in this study includes domains related to cognitive functioning, which reflect the patient’s subjective perception. This may partially overlap with the cognitive variables assessed separately; however, neuropsychological evaluation provides a more objective measure of cognitive functioning.

All participants underwent a standardized neurological examination and a comprehensive neuropsychological assessment conducted by trained clinicians.

Eligibility criteria included a diagnosis of RRMS, age between 18 and 70 years, and stable disease-modifying treatment for at least 3 months prior to enrollment. Patients with comorbid psychiatric disorders or other neurological conditions were excluded to ensure adequate cooperation during questionnaire administration, as well as to minimize potential confounding factors that could affect the reliability and interpretation of self-reported measures; also patients with language or visual impairments were excluded.

### 2.2 Outcome measures

Fatigue symptoms were assessed using the italian version of the Fatigue Severity Scale (FSS), a nine-item instrument with a 7-point Likert scale, where 1 represented strongly disagreement and 7 indicates strong agreement. The scale addresses fatigue’s effects on daily functioning, querying its relationship to motivation, physical activity, work, family, and social life (Krupp, 1989).

Fatigue was also assessed using the Modified Fatigue Impact Scale (MFIS), a self-report questionnaire that is the shortened version of the 40-item FIS questionnaire which has three dimensions and 21 questions. It is used to measure how fatigue affects physical, cognitive, and psychosocial functioning in individuals, (Kos et al., 2005).

Fatigue Scale for Motor and Cognitive Functions (FSMC), is a self-report questionnaire used to measure both physical (motor) and mental (cognitive). It consists of 20 items, with 10 items for cognitive fatigue and 10 for motor fatigue, scored on a 5 - point Likert scale, with total scores ranging from 20 to 100 (Penner et al., 2009).

The inclusion of three fatigue questionnaires was intentional, as they provide complementary information on fatigue in MS. The FSS was used to capture the global severity and functional burden of fatigue, the MFIS to assess the perceived impact of fatigue on physical, cognitive, and psychosocial functioning, and the FSMC to distinguish motor and cognitive fatigue more specifically.

Global disability was assessed by Expanded Disability Status Scale (EDSS) ranging from best to worst performance from 0 to 10 points (Kurtzke, 1983).

Cognitive level was measured by The Brief Repeatable Battery of Neuropsychological Tests (BRB-N). It consists of the Selective Reminding Test, the 10/36 Spatial Recall Test, the Symbol Digit Modalities Test, the Paced Auditory Serial Addition Test and the Word List Generation Test (Boringa et al., 2001).

Overall QoL was assessed by Multiple Sclerosis Quality of Life (MSQOL-54), a disease-specific instrument for individuals with MS. The composite instrument, composed of SF-36 and MS-18, is MSQOL-54 which contains 52 items grouped into 12 scales, plus two lone items. The instrument measures the anxiety provoked by one’s health status (four items), sexual functioning (four items), satisfaction with sex life (one item), overall QoL (two items), cognitive functioning (four items), energy/fatigue (one item), pain (one item), and social functioning (one item). The instrument consists of Likert scales and multiple-choice items (Solari et al., 1999).

### 2.3 Statistical analysis

The statistical analysis was conducted in a stepwise manner. In the first phase, analyses were performed on the entire sample to investigate the associations between fatigue-related measures (Fatigue, MFIS, FSMC, and FSS), cognitive performance (SDMT, PASAT, and SRT subtests of the Brief Repeatable Battery of Neuropsychological Tests), and selected domains of the MSQOL-54 (Health Perception, Role Physical Limitation, Role Emotional Limitation, Overall Quality of Life, and Cognitive Function). The Shapiro–Wilk test was used to assess the normality of the variables. Depending on the distribution of the data, either Pearson’s correlation coefficient (r) or Spearman’s rank correlation coefficient (*ρ*) was used to evaluate the relationships. Correlation analyses were corrected for multiple comparisons using the False Discovery Rate (FDR) method. Fatigue and neurological disability represent related but distinct aspects of MS. While EDSS reflects objective neurological impairment, fatigue could be subjective but also objective multidimensional symptom not directly proportional to disability. For this reason, both variables were included to explore their independent and combined contributions to cognitive functioning and QoL. To explore whether fatigue-related measures and neurological disability predicted cognitive performance and QoL outcomes, multiple linear regression analyses were performed. Full models included energy/fatigue, MFIS, FSMC, FSS, and neurological disability as measured by the EDSS as independent predictors.

A backward stepwise selection procedure was then applied, whereby non-significant predictors were sequentially removed until only variables significantly contributing to the explained variance of the dependent variable remained. Because the fatigue scales assess related but not identical constructs, multicollinearity was assessed in the full models using variance inflation factors (VIFs). VIF values indicated low collinearity for Fatigue and EDSS, whereas MFIS and FSS showed moderate collinearity, consistent with their partial conceptual overlap. Therefore, regression findings were interpreted with caution, particularly when considering the relative contribution of individual fatigue scales. This strategy was adopted to reduce model complexity and limit the risk of overfitting given the relatively small sample size. In the second phase, an exploratory subgroup analysis was performed by stratifying the sample according to disability severity using the EDSS score, distinguishing between a mild disability group (EDSS ≤5) and a moderate disability group (EDSS >5). Between-group comparisons were conducted to assess differences in fatigue measures, cognitive performance, and QoL outcomes. Independent-samples t-tests were applied when assumptions of normality and homogeneity of variance were satisfied; otherwise, Mann–Whitney *U* tests were used. Effect sizes were calculated for all between-group comparisons, using Cohen’s d for parametric tests and rank-biserial correlation (r) for non-parametric tests. Multiple comparisons were corrected using the Bonferroni method.

Finally, separate correlational analyses were performed within each disability subgroup using the same approach described above (Pearson or Spearman according to variable distribution). All tests were two-tailed, and statistical significance was set at *p* < 0.05. Statistical analyses were performed using R (version 4.4.2).

## 3 Results

### 3.1 Correlational analysis

The demographic and clinical characteristics of the sample, together with between-group comparisons according to disability level (EDSS ≤5 vs. EDSS >5), are reported in Table 1. Correlational findings for the whole sample are described below. Significant positive associations were observed between energy/fatigue and multiple QoL outcomes. Specifically, energy/fatigue was strongly correlated with Health Perception (*ρ* = 0.63, *p* < 0.001), Overall Quality of Life (*ρ* = 0.65, *p* < 0.001), Role Physical Limitation (*ρ* = 0.43, *p* = 0.002), and Role Emotional Limitation (*ρ* = 0.37, *p* = 0.007). After FDR correction, all four correlations remained statistically significant (*p*-FDR < 0.05), and associations with Health Perception and Overall QoL survived Bonferroni correction as well. The MFIS also showed significant negative correlations with SDMT (*r* = −0.38, *p* = 0.006), SRT-D (*r* = −0.36, *p* = 0.01), and Role Physical Limitation (*ρ* = −0.39, *p* = 0.004), with the first two remaining significant after FDR adjustment. MFIS was additionally associated with Overall QoL and Health Perception at uncorrected levels, but these did not survive correction. FSMC scores were negatively associated with SRT-CLTR (*r* = −0.31, *p* = 0.025), SDMT (*r* = −0.31, *p* = 0.03), and SRT-D (*r* = −0.39, *p* = 0.005); the latter remained significant after FDR correction (*p*-FDR = 0.03). Among fatigue-related measures, the FSS showed the strongest and most consistent associations. FSS correlated significantly with multiple QoL outcomes including Health Perception (*ρ* = −0.42, *p* = 0.002), Role Physical Limitation (ρ = −0.48, *p* < 0.001), Role Emotional Limitation (*ρ* = −0.33, *p* = 0.012), and Overall QoL (*ρ* = −0.39, *p* = 0.005), as well as cognitive variables such as SRT-LTS, SRT-CLTR, SDMT, and SRT-D (all *p* < 0.05). FSS–Role Physical Limitation remained significant even after Bonferroni correction (p_Bonf = 0.02), and several others retained significance after FDR correction. Lastly, EDSS exhibited a strong negative correlation with SDMT (*ρ* = −0.54, *p* < 0.001), which remained significant after Bonferroni corrections (p_Bonf = 0.003). Additional negative correlations were found between EDSS and Overall QoL (*ρ* = −0.32, *p* = 0.02), and a positive correlation was observed with Emotional Well-being (*ρ* = 0.29, *p* = 0.04), though these did not survive multiple comparison correction. Overall, the results support a consistent relationship between higher fatigue and disability levels and poorer cognitive functioning and perceived quality of life, particularly in processing speed and role-related domains.

**Table 1:** Between-group comparisons of fatigue, cognitive performance and perceived QoL in patients with MS, stratified by disability level (EDSS ≤5 vs. EDSS >5).

Variable	All sample	Group (EDSS ≤5)	Group (EDSS >5)	*p*	p Bonferroni	Effect size
		Mean ± SD	Mean ± SD			
Age	46.37 ± 12.39	39.14 ± 10.70	51.86 ± 10.76	<0.001^*^	0.004^*^	*r* = 0.52
Education (years)	5.98 ± 4.74	5.18 ± 4.49	6.59 ± 4.91	0.69	0.99	*r* = 0.06
EDSS	4.86 ± 1.18	3.68 ± 0.26	5.76 ± 0.69	<0.001^*^	< 0.001^*^	*r* = 0.86
Energy/fatigue	6.67 ± 5.15	8.0 ± 6.80	5.66 ± 3.21	0.32	0.51	*r* = 0.14
MFIS	45.94 ± 18.54	35.27 ± 17.74	54.03 ± 14.85	<0.001^*^	0.002^*^	*d* = −1.15
FSMC	69.08 ± 18.20	64.05 ± 20.58	72.90 ± 15.45	0.10	0.23	*d* = −1.15
FSS	45.55 ± 11.30	41.36 ± 12.35	48.72 ± 9.46	0.02^*^	0.14	*d* = 0.67
SRT-LTS	28.39 ± 14.40	29.13 ± 11.95	27.82 ± 16.20	0.74	0.78	*d* = 0.09
SRT-CLTR	22.19 ± 14.76	23.92 ± 13.01	20.87 ± 16.06	0.46	0.61	*d* = 0.21
SDMT	30.99 ± 13.51	39.05 ± 10.74	24.87 ± 12.23	<0.001^*^	0.001^*^	*d* = 1.23
PASAT-3	32.10 ± 14.81	34.22 ± 15.94	30.50 ± 13.96	0.39	0.57	*d* = 0.25
PASAT-2	27.45 ± 14.02	28.90 ± 13.54	26.35 ± 14.51	0.52	0.64	*d* = 0.18
SRT-D	6.94 ± 2.79	7.60 ± 2.49	6.45 ± 2.95	0.23	0.42	*r* = 0.17
Health perception	6.68 ± 4.14	7.98 ± 5.31	5.76 ± 2.71	0.10	0.23	*d* = 0.50
Role physical limitation	5.17 ± 4.06	6.30 ± 4.07	4.31 ± 3.90	0.07	0.23	*r* = 0.25
Role emotional limit	8.58 ± 6.64	8.02 ± 6.0	9.0 ± 7.16	0.78	0.78	*r* = 0.04
Overall quality life	7.78 ± 3.81	9.25 ± 4.04	6.67 ± 3.26	0.05	0.22	*r* = 0.27
Emotional well being	13.09 ± 6.87	11.46 ± 7.34	14.33 ± 6.33	0.15	0.30	*r* = 0.20
Cognitive_function	9.08 ± 4.45	9.33 ± 5.04	8.90 ± 4.03	0.75	0.78	*r* = 0.04

^*^p < 0.05.

Effect sizes are expressed as Cohen’s *d* for parametric tests and for non-parametric tests, effect size *r* was calculated as $Z/\sqrt{N}$ for non-parametric tests.

SD, standard deviation; EDSS, expanded disability status scale; MFIS, modified fatigue impact scale; FSMC, fatigue scale for motor and cognitive functions; FSS, fatigue severity scale; SRT-LTS, selective reminding test long-term storage; SRT-CLTR, selective reminding test consistent long-term retrieval; SRT-D, selective reminding test delayed recall; SDMT, symbol digit modalities test.

### 3.2 Multiple linear regression analysis

Multiple linear regression analyses using a backward stepwise procedure were conducted to identify independent predictors of cognitive and QoL outcomes. The final models retained only predictors that significantly contributed to each outcome variable. As reported in Table 2, EDSS emerged as a significant predictor of SDMT and Role Emotional Limitation. In parallel, the energy/fatigue score was a significant predictor of Health Perception, Overall QoL, Emotional Well-Being, and Cognitive Function. FSS significantly predicted Health Perception, Role Physical Limitation, and Role Emotional Limitation, whereas FSMC significantly predicted SRT-D and MFIS contributed to the prediction of Overall QoL. To assess the potential overlap among fatigue-related predictors, multicollinearity was examined in the full regression models using variance inflation factors (VIFs). VIF values were low for energy/fatigue (1.09) and EDSS (1.73), whereas FSMC (3.90), FSS (5.08), and MFIS (5.36) showed moderate collinearity, consistent with the partial conceptual overlap among self-report fatigue measures.

**Table 2:** Backward linear regression: significant predictors of each cognitive and QoL subscale.

Dependent	Predictor	Beta	Std error	*t* value	*p*-value	*R* ^2^	Adjusted *R*^2^	*p*-FDR
SRT-LTS	FSS	−0.40	0.17	−2.31	0.03	0.10	0.08	0.03
SRT-CLTR	FSS	−0.44	0.18	−2.50	0.02	0.11	0.09	0.02
SDMT	EDSS	−6.36	1.36	−4.67	<0.001	0.31	0.29	<0.001
PASAT-3	MFIS	−0.20	0.11	−1.80	0.08	0.06	0.04	0.08
SRT-D	FSMC	−0.06	0.02	−2.96	<0.01	0.15	0.13	0.01
Health perception	Energy/fatigue	0.48	0.08	5.88	<0.001	0.53	0.50	<0.001
	MFIS	0.07	0.04	1.54	0.13	0.53	0.50	0.13
	FSS	−0.19	0.07	−2.78	<0.01	0.53	0.50	0.01
Role physical limitation	FSS	−0.15	0.05	−3.31	<0.01	0.18	0.17	<0.01
Role emotional limitation	FSS	−0.25	0.08	−3.01	<0.01	0.20	0.16	0.01
	EDSS	2.22	0.81	2.75	<0.01	0.20	0.16	0.01
Overall quality of life	Energy/fatigue	0.37	0.09	4.36	<0.001	0.39	0.36	<0.001
	MFIS	−0.06	0.02	−2.43	0.02	0.39	0.36	0.02
Emotional well-being	Energy/fatigue	0.44	0.18	2.46	0.02	0.19	0.16	0.02
	EDSS	2.19	0.77	2.83	<0.01	0.19	0.16	0.01
Cognitive function	Energy/fatigue	0.31	0.12	2.70	<0.01	0.13	0.11	0.02

EDSS, expanded disability status scale; SDMT, symbol digit modalities test; FSS, fatigue severity scale.

### 3.3 Inter-group comparison

The inter-group analysis revealed significant differences in fatigue and cognitive performance between the mild (EDSS ≤5) and moderate (EDSS >5) disability groups (Table 1). Specifically, the moderate EDSS group exhibited significantly higher levels of fatigue as measured by the MFIS (*p* < 0.001, *d* = −1.15, p_Bonf = 0.004) and FSS (*p* = 0.02, *d* = −0.67, p_Bonf = 0.41) and significantly lower performance on the SDMT (*p* < 0.001, *d* = 1.23, p_Bonf < 0.001) compared to the mild group (Table 1). Among these, only the MFIS and SDMT results remained statistically significant after Bonferroni correction. While other comparisons did not reach statistical significance, several variables showed trends suggestive of group differences. Patients with moderate EDSS reported lower scores on Health Perception (*p* = 0.10), Overall Quality of Life (*p* = 0.05) and Role Physical Limitation (*p* = 0.07), with moderate effect sizes ranging from 0.25 to 0.50. No significant differences were found for verbal memory indices (SRT-LTS, SRT-CLTR, SRT-D), working memory (PASAT-2 and PASAT-3), emotional well-being, and global cognitive self-assessment.

### 3.4 Correlation analysis in the mild disability group (EDSS ≤5)

**Figure 1:**
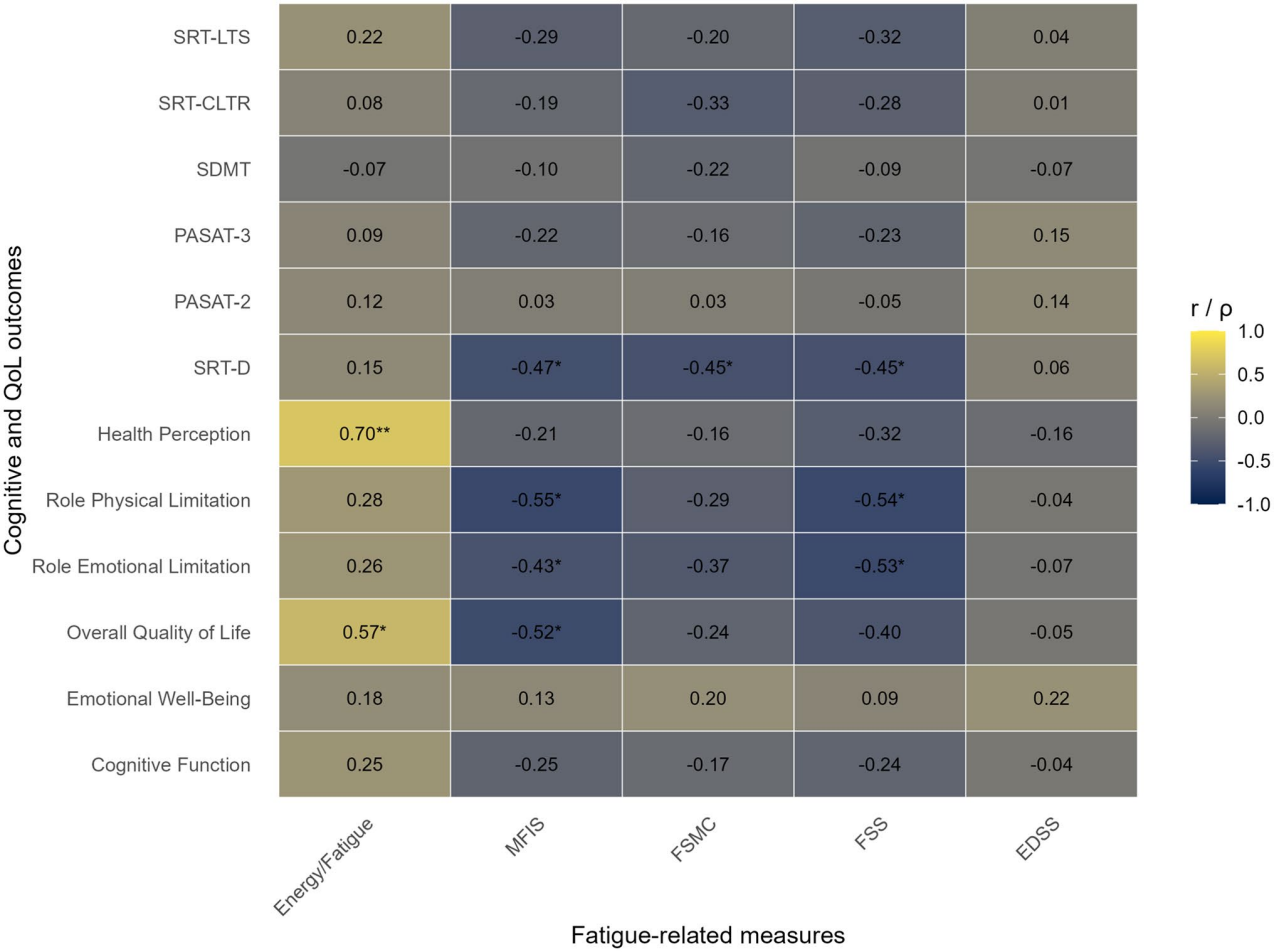
Correlation matrix for the mild disability group (EDSS ≤5). Correlation coefficients between fatigue-related measures (Energy/fatigue, MFIS, FSMC, FSS, and EDSS) and cognitive/QoL outcomes in patients with mild disability. Cell color reflects the strength and direction of the association. Values within cells represent Pearson’s *r* or Spearman’s *ρ*, according to variable distribution. *indicates *p* < 0.05; **indicates *p*-FDR <0.05.

In the group of individuals with mild neurological disability, several significant associations emerged between fatigue measures and domains of perceived QoL (Figure 1). The general fatigue score showed a strong positive correlation with Health Perception (*ρ* = 0.70, *p* = 0.0003), which remained significant after both FDR correction (*p*-FDR = 0.01), indicating a robust relationship between perceived fatigue and physical self-assessed health even in patients with lower disability levels. Additionally, a moderate correlation was observed with Overall QoL (*ρ* = 0.57, *p* = 0.006), which was significant at the uncorrected level but did no remain significant after FDR correction (*p*-FDR = 0.10). Among the fatigue subscales, MFIS was negatively associated with SRT-D (*r* = −0.47, *p* = 0.03), Role Physical Limitation (ρ = −0.55, *p* = 0.008) and with Overall QoL (*ρ* = −0.52, *p* = 0.013), but these results did not survive correction for multiple comparisons. A weaker association was also observed between MFIS and Role Emotional Limitation (*r* = −0.43, *p* = 0.044). FSMC showed a moderate negative correlation with SRT-D (*r* = −0.45, *p* = 0.03), indicating that higher fatigue severity was associated with lower verbal memory performance. FSS highlighted significant negative correlations with several QoL domains. In particular, FSS was negatively associated with Role Physical Limitation (*ρ* = −0.54, *p* = 0.01) and Role Emotional Limitation (*r* = −0.53, *p* = 0.011). FSS was also negatively associated with SRT-D (*r* = −0.45, *p* = 0.04), indicating the link between fatigue and verbal episodic memory deficits. Overall, these findings suggest that even in patients with mild disability, fatigue is meaningfully associated with reduced quality of life and, to a lesser extent, with specific cognitive measures such as verbal memory performance.

### 3.5 Correlation analysis in the moderate disability group (EDSS >5)

**Figure 2:**
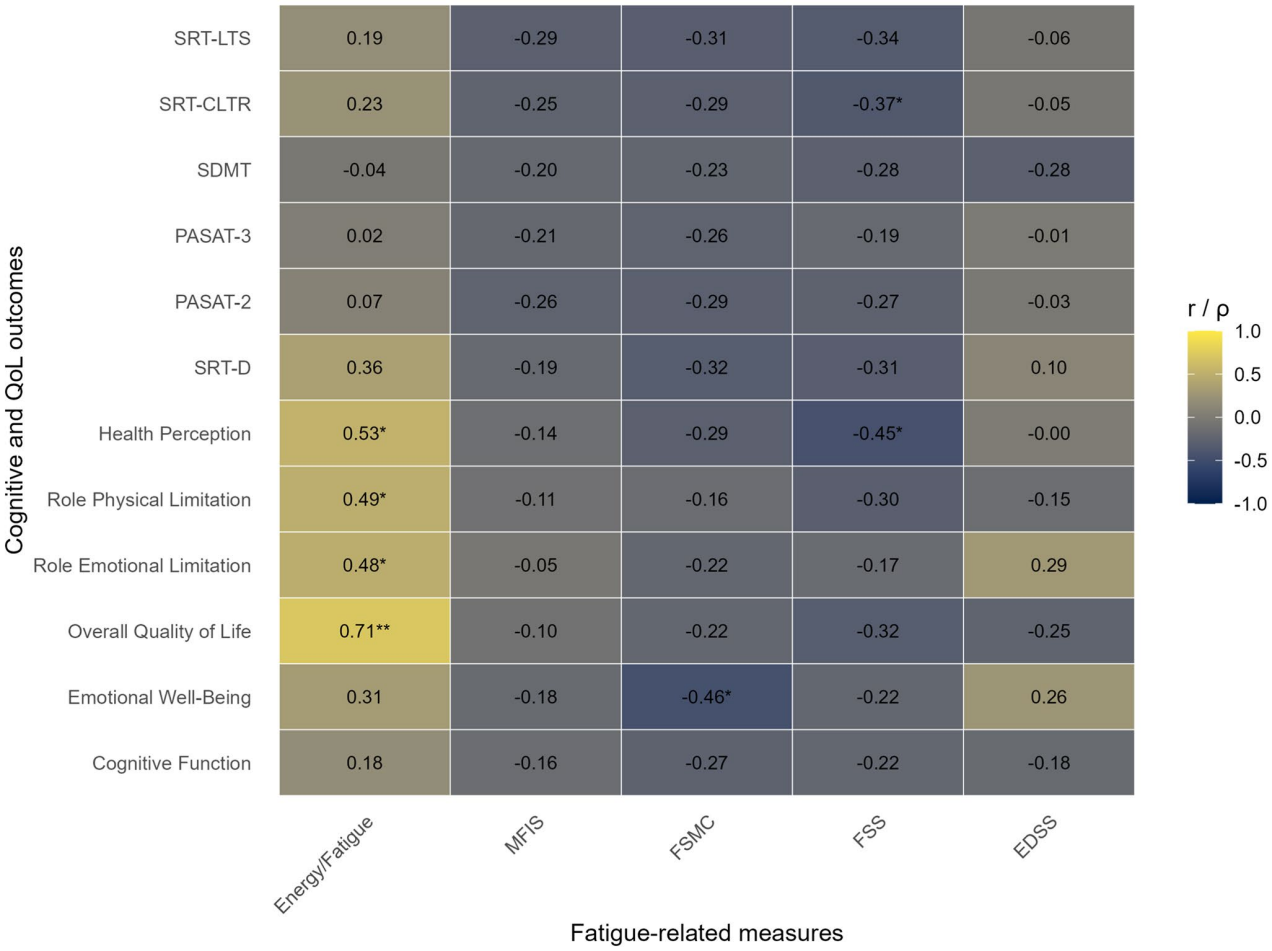
Correlation matrix for the moderate disability group (EDSS >5). Correlation coefficients between fatigue-related measures (Energy/fatigue, MFIS, FSMC, FSS, and EDSS) and cognitive/QoL outcomes in patients with moderate disability. Cell color reflects the strength and direction of the association. Values within cells represent Pearson’s *r or* Spearman’s *ρ*, according to variable distribution. *Indicates *p* < 0.05; **Indicates *p*-FDR <0.05.

Within the group of participants with moderate disability, several correlations emerged between fatigue scales and measures of QoL, although most did not remain significant after Bonferroni or FDR correction (Figure 2). The general fatigue score showed strong positive associations with Health Perception (*ρ* = 0.53, *p* = 0.003), role physical limitation (*ρ* = 0.49, *p* = 0.007), and Role Emotional Limitation (*ρ* = 0.48, *p* = 0.008), as well as a very strong association with overall QoL (*ρ* = 0.71, *p* < 0.001), which remained significant after correction (*p*-FDR < 0.001), suggesting a robust relationship between perceived fatigue and overall QoL in this subgroup. FSMC also showed a moderate negative correlation with Emotional Well-Being (*ρ* = −0.46, *p* = 0.01) and Health Perception (*r* = −0.45, *p* = 0.01), indicating that higher fatigue related complaints were associated with poorer emotional and physical self-perceived health. However, these results did not survive correction for multiple comparisons. Finally, a trend was observed between the FSS and the SRT-CLTR (*r* = −0.37, *p* = 0.05), suggesting a possible link between fatigue severity and verbal learning, although this also did not remain significant after correction.

## 4 Discussion

The present study aimed to investigate whether fatigue is associated with cognitive impairment and overall QoL in individuals with MS. Overall, our findings indicate that fatigue is strongly associated with several domains of QoL, particularly physical and emotional well-being, and is also related to specific aspects of cognitive functioning. These results contribute to a more comprehensive understanding of disease burden in MS, highlighting fatigue as a key factor that interacts with multiple dimensions of patient functioning. One of the main findings of this study is the robust association between fatigue and perceived QoL. Higher levels of fatigue were consistently related to poorer health perception, greater physical and emotional role limitations, and lower overall QoL. These findings emphasize that fatigue substantially influences patients’ subjective experience of the disease and is strongly associated with QoL outcomes. Notably, fatigue remained a significant predictor of several QoL domains showing robust associations in patients with lower disability levels, suggesting that the impact was extended beyond neurological disability. The observed associations between fatigue and both physical and emotional limitations support the view that fatigue in MS is not merely a physical symptom, but a complex phenomenon that also affects psychological well-being and emotional functioning. Previous research has highlighted the role of affective factors, particularly depressive symptoms, in shaping the relationship between fatigue and QoL (Hu et al., 2019; Wolkorte et al., 2015), suggesting that fatigue is embedded within a broader psychosocial context.

Regarding cognitive functioning, our results align with previous literature reporting heterogeneous and sometimes inconsistent associations between perceived fatigue and objective cognitive performance in MS (Bol et al., 2010; Taranu et al., 2025). In the present study, higher levels of perceived fatigue, as measured by the MFIS, FSMC, and FSS, were associated with poorer performance on the SDMT and the delayed recall component of the SRT-D, reflecting deficits in processing speed and verbal episodic memory. Processing speed is one of the most frequently impaired cognitive domains in MS and plays a crucial role in daily functioning. The association between fatigue and SDMT performance suggests that fatigue may particularly affect cognitive efficiency and the ability to sustain rapid information processing. Similarly, the association with delayed verbal recall indicates that fatigue may interfere with memory-related processes, which are commonly compromised in MS (Gich et al., 2024). Notably, among the fatigue measures employed, the FSS demonstrated the strongest and most consistent associations with both cognitive outcomes and QoL domains. This finding suggests that more global and subjective perceptions of fatigue may be especially sensitive to capturing the overall clinical burden experienced by patients. The association between FSS scores and episodic verbal memory performance is particularly relevant, as it supports the hypothesis that broader fatigue experiences may reflect an integration of physical, cognitive, and emotional difficulties rather than isolated functional impairments. In addition, the strong associations between FSS and physical and emotional role limitations further reinforce the interconnected nature of fatigue, cognition, and emotional well-being. Disability level, as assessed by the EDSS, also played a significant role in shaping fatigue and cognitive performance. Comparisons between patients with mild (EDSS ≤5) and moderate disability (EDSS >5) revealed that individuals with greater neurological impairment reported higher fatigue levels and showed poorer cognitive performance, particularly in processing speed. Specifically, patients with moderate disability exhibited significantly higher MFIS and FSS scores and significantly lower SDMT performance. These findings are consistent with previous studies indicating that fatigue tends to increase with neurological disability, while cognitive efficiency declines, especially in tasks requiring rapid cognitive processing. Importantly, despite increasing physical disability, fatigue continued to exert a substantial impact on QoL among patients with moderate disability. Lower scores in perceived health and overall QoL in this group suggest that fatigue remains a central determinant of subjective well-being even in more advanced stages of MS. This highlights the clinical relevance of fatigue across different levels of disability and underscores the need for targeted fatigue management strategies throughout the disease course. Taken together, these findings support the conceptualization of fatigue in MS as a multidimensional construct in which physical, cognitive, and emotional components interact and reinforce one another. Rather than acting as an isolated symptom, fatigue appears to function as a transversal factor that may amplify the impact of other disease-related impairments, including cognitive dysfunction and emotional distress. While earlier studies often conceptualized fatigue primarily as a physical phenomenon with limited cognitive correlates, our results suggest that subjective fatigue experiences—particularly those captured by global measures such as the FSS—are meaningfully associated with both cognitive performance and QoL outcomes. The co-occurrence of fatigue, emotional limitations, physical dysfunction, and cognitive slowing may therefore act synergistically to worsen patients’ perceived QoL (Guillemin et al., 2022). Several limitations should be considered when interpreting the present findings. First, the cross-sectional design precludes any causal inference regarding the directionality of the relationships between fatigue, cognitive impairment, and QoL. Longitudinal studies are needed to determine whether fatigue precedes cognitive decline or represents a consequence of cognitive and psychosocial changes. Second, the relatively small sample size, the absence of the sample size calculation and single-center recruitment may limit the generalizability of the results. In addition, because the EDSS-based subgroup analyses were conducted on smaller samples, these findings should be regarded as exploratory and interpreted with caution.

Additionally, the relatively small sample size may increase the risk of overfitting in regression analyses, although the use of backward stepwise selection allowed us to limit model complexity and retain only significant predictors. An additional methodological consideration concerns the use of multiple self-report fatigue measures. Although the FSS, MFIS, and FSMC were selected to capture complementary dimensions of fatigue, multicollinearity analyses indicated moderate overlap between some instruments, particularly MFIS and FSS. Accordingly, the multivariable models should be interpreted as identifying the relative contribution of partially overlapping fatigue dimensions, rather than as demonstrating fully independent effects of entirely distinct constructs.

Third, depressive symptoms were not formally assessed with a specific standardized questionnaire, which is particularly relevant given the well-established overlap between fatigue and depression in MS. As a result, the extent to which the observed associations between fatigue, cognitive outcomes, and QoL reflect fatigue per se, rather than partially overlapping affective symptoms, cannot be fully determined. This is particularly important for outcomes related to emotional well-being and subjective QoL, which may be especially sensitive to mood-related influences. Future studies should include standardized measures of depression and anxiety in order to better disentangle the specific contribution of fatigue from broader affective burden. Finally, although multiple fatigue instruments were used, the reliance on self-report measures may have introduced subjective bias. Future studies should integrate objective markers of fatigability and neuroimaging correlates to further clarify underlying mechanisms. Despite these limitations, the main strength is that the assessment of fatigue was conducted in a relatively homogeneous cohort of patients with relapsing–remitting multiple sclerosis receiving stable treatment, which enhances the internal validity of the findings by limiting heterogeneity related to disease course and therapeutic fluctuations that may influence fatigue perception. Moreover, the present findings have important clinical implications. They emphasize the need to systematically assess fatigue in routine MS care and to consider its multidimensional nature when planning interventions. Therapeutic approaches targeting both physical and emotional components of fatigue may not only improve QoL but also have beneficial effects on cognitive functioning. Integrating fatigue management into comprehensive MS rehabilitation programs may therefore represent a valuable strategy to reduce overall disease burden and enhance patients’ daily functioning.

## 5 Conclusion

In conclusion, this study indicates that fatigue is a key determinant of disease burden in individuals with multiple sclerosis, with a substantial impact on QoL and selective cognitive domains. Higher fatigue levels were associated with poorer health perception, greater physical and emotional role limitations, and reduced overall QoL, as well as lower performance in processing speed and verbal episodic memory. These findings support the multidimensional nature of fatigue, encompassing physical, cognitive, and emotional components, and highlight its relevance across different levels of neurological disability. Although causal inferences cannot be drawn due to the cross-sectional design, the results underscore the importance of systematically assessing and managing fatigue in clinical practice. Beyond confirming the clinical relevance of fatigue in MS, the present study adds to existing knowledge by showing, within a homogeneous RRMS cohort, that fatigue is linked to both QoL and selected cognitive domains when examined through a multidimensional assessment framework and across different levels of neurological disability. Targeted interventions addressing fatigue may contribute to improved well-being and cognitive efficiency in people with MS.

## Data Availability

The raw data supporting the conclusions of this article will be made available by the authors, without undue reservation.
